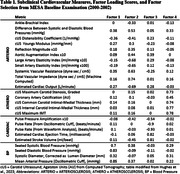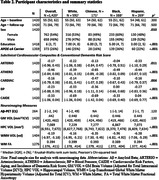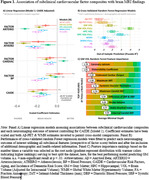# Subclinical Vascular Risk Composites and Dementia Imaging Biomarkers 17‐20 years later in the Multi‐Ethnic Study of Atherosclerosis (MESA)

**DOI:** 10.1002/alz.089603

**Published:** 2025-01-09

**Authors:** Marc D. Rudolph, Jordan E. Tanley, Jingzhong Ding, Haiying Chen, Kathleen M. Hayden, Yongmei Liu, R. Nick Bryan, Ilya M. Nasrallah, Sudipto Dolui, Mohamad Habes, José A. Luchsinger, Susan R. Heckbert, Samuel N. Lockhart, Timothy M. Hughes

**Affiliations:** ^1^ Wake Forest University School of Medicine, Winston‐Salem, NC USA; ^2^ Wake Forest University School of Medicine, Winston Salem, NC USA; ^3^ University of Pennsylvania, Philadelphia, PA USA; ^4^ Department of Radiology, University of Pennsylvania, Philadelphia, PA USA; ^5^ Glenn Biggs Institute for Alzheimer’s & Neurodegenerative Diseases, University of Texas Health Sciences Center at San Antonio, San Antonio, TX USA; ^6^ Columbia University Irving Medical Center, New York, NY USA; ^7^ University of Washington, Seattle, WA USA

## Abstract

**Background:**

Vascular risk factors captured in midlife represent modifiable features of cardiovascular disease (CVD), stroke, dementia, and dementia‐related neuropathology. Subclinical measures of CVD may help identify specific structural and function aspects underlying vascular contributions to cognitive impairment and dementia over and above conventional dementia risk scores.

**Method:**

The MESA study followed a diverse cohort of 6,814 adults aged 45‐84 years over 6 clinical examinations and annual follow‐up calls since baseline, 2000‐2002. We evaluated relationships between *subclinical* cardiovascular assessments collected at baseline (Table 1) and neuroimaging biomarkers (Table 2) acquired ∼17‐20 years later in a subset (N = 1420) of older adults free from clinical CVD at baseline with MRI data (Table 2). Factor analyses identified four subclinical composites of arteriosclerosis, atherosclerosis, cardiac function, and blood pressure. Cardiovascular Risk Factors, Aging, and Incidence of Dementia Risk (CAIDE) composite scores comprised of age, sex, education, systolic blood pressure, body‐mass index, total cholesterol, physical activity and *APOE*‐ε4 genotype were calculated. Adjusted general linear models and Random Forest modeling paired 5‐fold 10x‐repeated cross‐validation were used in all analyses.

**Results:**

Baseline subclinical factors were differentially associated with MRI measures at long‐term follow‐up. Subclinical composites of arteriosclerosis and atherosclerosis exhibited the strongest associations with lower global and hippocampal gray matter volume, greater white matter hyperintensity burden, and with higher Aβ‐PET deposition in unadjusted (not shown) and adjusted models (Figure 1, panel A; all *p* < .05). A composite of cardiac function was modestly associated with MRI measures. Blood pressure was more strongly associated with measures of white matter brain health. Cross‐validated prediction models utilizing individual subclinical measures best predicted measures of global and regional GM volume (Figure 1, panel B). Subclinical measures representing arteriosclerosis (e.g., small artery elasticity, systemic vascular resistance, carotid distensibility) and atherosclerosis (e.g., common carotid intimal‐media thickness, maximum intimal‐media thickness) were most predictive across imaging outcomes assessed. Baseline coronary calcification and carotid distensibility were most strongly associated with MRI measures indexing global GM atrophy (Figure 1, panel C).

**Conclusion:**

Ultimately, our findings may highlight specific subclinical vascular pathways to dementia. Extending this work, we aim to develop trajectory‐based prediction models to better assess vascular aging.